# Transcriptional organization and regulation of the *Pseudomonas putida* K1 type VI secretion system gene cluster

**DOI:** 10.1099/mic.0.001295

**Published:** 2023-01-27

**Authors:** Patricia Bernal, Cristina Civantos, Daniel Pacheco-Sánchez, José M. Quesada, Alain Filloux, María A. Llamas

**Affiliations:** ^1^​ Department of Environmental Protection, Estación Experimental del Zaidín (CSIC), Granada, Spain; ^2^​ MRC Centre for Molecular Bacteriology and Infection, Department of Life Sciences, Imperial College London, London, UK; ^3^​ Departamento de Microbiología, Facultad de Biología, Universidad de Sevilla, 41012 Seville, Spain; ^4^​ Singapore Centre for Environmental Life Sciences Engineering. Nanyang Technological University, Singapore

**Keywords:** type VI secretion system, gene regulation, RetS, GacS–GacA, RpoN, RpoS, *Pseudomonas*, FleQ

## Abstract

The type VI secretion system (T6SS) is an antimicrobial molecular weapon that is widespread in Proteobacteria and offers competitive advantages to T6SS-positive micro-organisms. Three T6SSs have recently been described in *Pseudomonas putida* KT2440 and it has been shown that one, K1-T6SS, is used to outcompete a wide range of phytopathogens, protecting plants from pathogen infections. Given the relevance of this system as a powerful and innovative mechanism of biological control, it is critical to understand the processes that govern its expression. Here, we experimentally defined two transcriptional units in the K1-T6SS cluster. One encodes the structural components of the system and is transcribed from two adjacent promoters. The other encodes two hypothetical proteins, the tip of the system and the associated adapters, and effectors and cognate immunity proteins, and it is also transcribed from two adjacent promoters. The four identified promoters contain the typical features of σ*^70^*-dependent promoters. We have studied the expression of the system under different conditions and in a number of mutants lacking global regulators. *P. putida* K1-T6SS expression is induced in the stationary phase, but its transcription does not depend on the stationary σ factor RpoS. In fact, the expression of the system is indirectly repressed by RpoS. Furthermore, it is also repressed by RpoN and the transcriptional regulator FleQ, an enhancer-binding protein typically acting in conjunction with RpoN. Importantly, expression of the K1-T6SS gene cluster is positively regulated by the GacS–GacA two-component regulatory system (TCS) and repressed by the RetS sensor kinase, which inhibits this TCS. Our findings identified a complex regulatory network that governs T6SS expression in general and *P. putida* K1-T6SS in particular, with implications for controlling and manipulating a bacterial agent that is highly relevant in biological control.

## Introduction

The type VI secretion system (T6SS) is a bacterial contractile nanomachine found in approximately 25 % of Gram-negative bacteria. The T6SS is used to deliver effectors/toxins into neighbouring cells in a contact-dependent manner. Since its discovery in 2006, most T6SS studies have been performed in bacterial human pathogens such as *Pseudomonas aeruginosa *, *Vibrio cholerae* and enteropathogenic *Escherichia coli* strains, and it was initially assumed that T6SS’s main role was in delivering effectors inside eukaryotic cells [1–3]. However, more recent studies revealed that most T6SS effectors are antibacterial toxins and are produced and secreted by both pathogenic and non-pathogenic bacteria [4]. It is now generally recognized that the T6SS is important for interbacterial competition and that in non-pathogenic environmental bacteria this system is mainly used to outcompete foes [5–7].

**Fig. 1.**
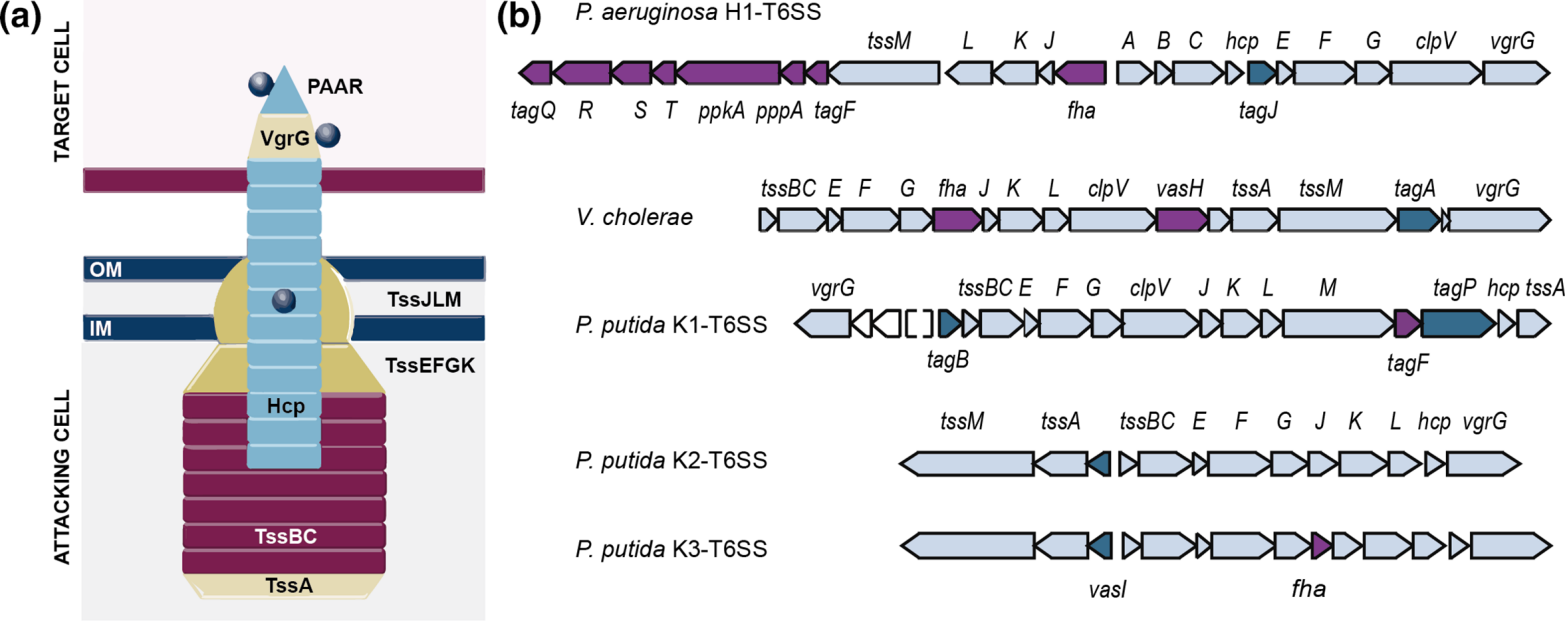
The T6SS apparatus. (**a**) Schematic representation of a T6SS after being fired. The VgrG/PAAR tip complex (beige/cyan), the Hcp tube (cyan) and the coupled effectors (grey) are ejected from the attacker into a target cell (pink). The membrane complex (TssJLM) and baseplate (TssEFGK) structures are shown in gold, and the sheath (TssBC) and the cap protein TssA are coloured in dark red and beige, respectively. (**b**) Schematic representation of the genomic organization of the structural genes of the H1-T6SS cluster from *P. aeruginosa *, *V. cholerae* and the K1-, K2- and K3-T6SS clusters from *P. putida *. Genes encoding core components are shown in light blue, accessory components are depicted in dark blue and regulatory elements are coloured in purple.

The T6SS is made up of 12 core components that form a membrane complex (TssJLM) that anchors the system to the cell envelope and holds a baseplate platform (TssEFGK). From this platform and primed by a ring of TssA proteins, a tail polymerizes from the tip (VgrG and PAAR) and is followed by an inner tube of Hcp rings wrapped within a contractile sheath of TssBC proteins [8] (Fig. 1a). At the structural level, these proteins and the ATPase that recycles the sheath (ClpV) are highly conserved among the T6SS clusters, with few exceptions. Despite the general conservation of the structural components of the apparatus, different phylogenetic groups have been described [9, 10] and the T6SS clusters have been shown to differ in the organization of the genes encoding the core components (Fig. 1b) [10], the presence of genes encoding a variable number of accessory proteins (TagP, TagJ, TagL, TagA, TagB) [11–13] (Fig. 1b, dark blue), genes encoding effector–immunity pairs and, more importantly, genes encoding specific regulatory elements that control the T6SS at different levels (Fig. 1b, purple).

The activity of the T6SSs is tightly regulated at each possible stage, from transcriptional to translational and post-translational levels. It is common to find genes that encode T6SS regulatory elements within the T6SS clusters, with the most common elements being PpkA/PppA serine–threonine kinase/phosphatase systems, bacterial enhancer-binding proteins (bEBPs) and other regulators, such as TagF and forkhead-associated (FHA) domain-containing proteins [14–18]. For example, the *P. aeruginosa* H1-T6SS cluster encodes seven different regulatory components, including *tagF1*, *pppA*, *ppkA* and *tagQRST* [19–21] (Fig. 1b, purple). The PpkA/PppA system regulates the activity of the *P. aeruginosa* H1-T6SS at the post-translational level by phosphorylating/dephosphorylating an FHA domain-containing protein termed FHA1 that is required for the assembly of the H1-T6SS and the secretion of Hcp1 [14]. The accessory proteins TagOPQRST form a transmembrane signalling pathway that acts upstream of the PpkA/PppA, ultimately activating the H1-T6SS under surface growth conditions [19, 20]. This additional level of regulation is not widespread, but it can be found in other species of *Pseudomonas* such as *P. fluorescens* [6]. Proteins containing the FHA domain are found encoded in many other T6SS clusters, including those from *Serratia marcescens* [22] and *Agrobacterium tumefaciens* [23]. In *A. tumefaciens *, FHA interacts with a phosphorylated (*p*-) membrane complex component *p*-TssL, a process crucial for the interaction of TssL with Hcp and activation of type VI subassembly and secretion [23]. The phosphorylation-independent pathway of *P. aeruginosa* H1-T6SS is governed by TagF [24], a post-translational repressor that can be found in many other T6SS clusters (Fig. 1b). In *P. aeruginosa *, *A. tumefaciens* and *S. marcescens *, TagF interacts with *p-*FHA, impacting on T6SS assembly [16, 25].

T6SSs are also regulated by global regulatory mechanisms, including quorum sensing (QS), two-component systems (TCSs), histone-like proteins (H-NS), transcriptional factors (TFs), σ factors and small regulatory RNAs [26]. These global regulatory mechanisms control the expression of many processes in the cell and are widespread in bacteria. Importantly, although T6SS clusters have been horizontally transferred, the production of these systems has been incorporated into former regulatory networks. This observation suggests that these master regulators will allow coordination of T6SS expression, assembly and secretion, necessary to outcompete foes, with other important and equally energetically expensive processes to minimize energy costs. QS, which controls gene expression in response to bacterial cell population, is a major regulatory pathway for T6SS gene expression [26]. In *P. aeruginosa *, production of the three T6SSs of this bacterium is controlled by the LasRI and the MvfR (also known as PqsR) QS systems [27], whereas the CviR QS regulator controls the expression of the *Chromobacterium violaceum* T6SS cluster [28]. Furthermore, the most common regulatory mechanism governing the T6SS expression is TCSs. TCSs couple the sensing of an environmental signal to an adaptive response by a sensor kinase and a response regulator, allowing bacteria to adapt to different environmental conditions. Many T6SS loci are regulated by the GacS–GacA TCS, which is additionally modulated by other sensor kinases, such as RetS and/or LadS [29]. This signalling cascade converges on small RNAs (e.g. *rsmZ* and *rsmY* in *P. aeruginosa *), which control the function of RNA-binding proteins (e.g. RsmA) that often act as translational repressors of T6SSs [30]. In several bacteria, histone-like proteins repress the transcription of T6SS genes by their general mechanism, i.e*.* binding to AT-rich regions. For instance, MvaT, a *P. aeruginosa* H-NS-like protein, represses the H2- and H3-T6SS loci [31] and the TurA H-NS protein seems to silence the *P. putida* K2-T6SS [32]. Transcriptional factors that function as global regulators, such as the ferric uptake regulator (Fur), have also been involved in the regulation of T6SS loci [26, 33]. Moreover, although transcription of most T6SS genes depends on the housekeeping sigma factor σ^70^ (also known as RpoD or σ^A^), other sigma factors modulate transcription of T6SS loci. This includes the alternative σ factor RpoS (also known as σ^38^ or σ^S^), which belongs to the σ^70^ family of σ factors. RpoS regulates the bacterial general stress response and is activated in the stationary phase of growth [34]. Although RpoS does not appear to be a major regulatory player for T6SS expression, it has been shown to positively regulate the *Klebsiella pneumoniae* and *Yersinia pseudotuberculosis* T6SS loci [35, 36]. In the plant pathogen *Xanthomonas citri,* an alternative σ factor of the extracytoplasmic function sigma (σ^ECF^) factor group [37], the σ^EcfK^ factor, promotes T6SS expression during the interaction of the bacterium with its amoeba predator, *Dictyostelium* [38]. Further, RpoN (also known as σ^N^ or σ^54^), which is the only member of a different family of σ factors, the σ^54^ family [39], also modulates T6SS expression [26]. RpoN-dependent promoters require bacterial enhancer-binding proteins (bEBPs) to activate transcription initiation [40]. Interestingly, genes encoding these proteins are often found within the T6SS clusters. For example, *vasH* in the *V. cholerae* T6SS (Fig. 1b) [2, 18, 41] and *sfa2* and *sfa3* encoded in the *P. aeruginosa* H2 and H3-T6SSs, respectively [15]. Curiously, the bEBP FleQ, which is a master regulator of flagella biosynthesis, has been associated with T6SS regulation in different species of *Pseudomonas* [42–44].

The biocontrol agent *P. putida* KT2440, an environmental strain able to promote the growth of crop plants and defend plants from the attack of phytopathogens, contains three T6SS gene clusters [5, 45, 46] (Fig. 1b). We have recently reported that the K1-T6SS of this bacterium is an effective mechanism of biocontrol. K1-T6SS is a powerful molecular weapon used by *P. putida* to outcompete plant pathogens, including *A. tumefaciens *, *Pseudomonas syringae *, *Xanthomonas campestris *, *Pectobacterium carotovorum* and *Pseudomonas savastanoi *, among others [5, 11]. The structural components and associated effectors of the *P. putida* K1-T6SS have been studied in previous works [5, 10, 11]; however, the regulation of the system is still unknown. In this work, we have studied the transcriptional regulatory network that governs the *P. putida* K1-T6SS cluster. We have identified two transcriptional units within the K1-T6SS locus and four σ^70^-dependent promoters driving transcription of the K1-T6SS operons. We found that the expression of the system is induced at the stationary phase of growth by a still unknown mechanism that does not involve the stationary phase σ factor RpoS, which represses instead of induces expression of these genes. Analysis of K1-T6SS expression in several global regulators mutants showed that the system is also repressed by RpoN and its activator FleQ. A potential RpoN-binding box was found in the K1-T6SS promoter. However, mutation of this box showed that RpoN did not bind to this site. On the contrary, the K1-T6SS expression is activated by the GacS–GacA TCS, while its associated sensor protein RetS represses transcription of the system. Our results show that production of the *P. putida* K1-T6SS is governed by a complex regulatory network that in this way manages the biocontrol capabilities of this bacterium.

## Methods

### Bacterial strains and growth conditions

Bacterial strains are listed in Table S1 (available with the online version of this article). Unless otherwise stated, chemicals and reagents, including antibiotics, were purchased from Sigma-Aldrich and components to prepare growth media were purchased from Oxoid. All strains were grown in lysogeny broth (LB) (10 g l^−1^ NaCl) and agar (1.5 % w/v) [47] for routine growth with shaking at 200 r.p.m., as appropriate. *E. coli* strains were incubated at 37 °C and *P. putida* strains at 30 °C. Antibiotics were used at (µg ml^−1^): rifampicin (Rif), 20 for *Pseudomonas *; tetracycline (Tc), 20 for *P. putida* and 10 for *E. coli *; kanamycin (Km), 50 for *P. putida* and 25 for *E. coli *; ampicillin (Ap) 100 for *E. coli *; piperacillin (Pip), 25 for *P. putida *.

### Plasmids and cloning

Plasmids and primers used in this study are listed in Tables S2 and S3. DNA manipulations were performed using standard methods [47].

KOD Hot Start DNA polymerase (Merck) or Phusion High-Fidelity DNA polymerase (New England Biolabs) were used for PCR reactions according to the manufacturer’s instructions. Primers were synthesized by Sigma-Aldrich and restriction enzymes were purchased from New England Biolabs. All DNA constructs were sequenced and verified to be correct before use.

The 343, 507 and 456 bp DNA regions containing the putative K1-T6SS promoter regions P*_tagB1_,* P*_hcp1_* and P*_vrgG1_*, respectively, were amplified from genomic DNA extracted from the *P. putida* KT2440 strain using primers P1–P6 (Table S3). Promoters were cloned into the broad-host-range, low-copy-number vector pMP220 at the EcoRI/KpnI/PstI sites to produce transcriptional fusions to the *lacZ* gene. Recombinant plasmids were sequenced and transferred to *P. putida* by electroporation [48].

A DNA region upstream of PP3084 (173 bp) was amplified from genomic DNA extracted from the *P. putida* KT2440 strain using primers P7–P8 (Table S3) and cloned into pMP220 at EcoRI/XbaI sites to produce a transcriptional fusion to the *lacZ* gene. The gene PP3086 and its promoter region (711 bp) were amplified using primers P9–P10 (Table S3) and cloned into pMMB67EH at the EcoRI/HindIII sites. Recombinant plasmids (pMP220-based and pMMB67_PP3086) were sequenced and co-transferred to *P. putida* by electroporation [48].

### β-galactosidase assays

Overnight cultures of the *P. putida* strains carrying the pMP220 plasmid and derivates were diluted to a final turbidity (A_600_) of 0.05 in fresh LB medium containing tetracycline and cultures were grown at 30 °C and 200 r.p.m. for 4 and 24 h. Turbidity reached around 0.7 after 4 h and 5.5 after 24 h, and at this point aliquots were taken to measure *β*-galactosidase activity in permeabilized whole cells as described by Miller [49]. At least three independent assays were performed in each case, and standard errors of the means were calculated.

*The P. putida* strains co-transformed with the plasmids derived from pMP220 and pMMB67 were handled in the same manner and the samples were prepared as explained above (exponential phase of growth).

### RNA purification

*P. putida* KT2440 was grown at 30 °C in LB at 200 r.p.m. until turbidity reached approximately 0.7 (exponential phase cultures) or 5–6 (stationary phase cultures). Cells were harvested by centrifugation (8,000 ***g*** for 5 min at 4 °C) in disposable plastic tubes precooled in liquid nitrogen and the pellets were kept at −80 °C until use. Total RNA was extracted using the TRI reagent method (Ambion, ref. 9738, Austin, TX, USA) as recommended by the manufacturer, except that TriPure Isolation Reagent was preheated at 70 °C followed by purification with RNeasy columns (Qiagen). A final digestion step with RNase-free Ambion TURBO DNase was added at the end of the process. The RNA concentration was determined using a spectrophotometer and RNA integrity was assessed by 2 % agarose gel electrophoresis.

### Reverse transcription-PCR

To determine the transcriptional unit of the K1-T6SS cluster, reverse transcription PCR was performed as previously described by Duque *et al*. [50] with primers P11–P36 (Table S3) based on adjacent genes. When the genes have a small size, the primers cover the intergenic regions between three adjacent genes to obtain an optimal PCR product in the range of 0.5 to 1.5 kb. We have used mRNA isolated from *P. putida* KT2440 cells in the stationary phase of growth as indicated above and the Roche One-Step RT-PCR kit.

### Quantitative real-time PCR

Total RNA was retrotranscribed to cDNA with Invitrogen Superscript II reverse transcriptase using random hexamers as primers. The specific primer pairs used in this study to amplify cDNA were: P37–P38 for the gene encoding Hcp1; P39–P40 for the gene encoding TssB1; P41–P42 for the gene encoding PP3084; and P43–P44 for the gene encoding 16S rRNA (Table S3). Real-time PCR was performed using the iCycler iQ Real-Time PCR Detection System (Bio-Rad). The target cDNA of the experimental and reference samples was amplified in triplicate.

Each 25 µl reaction contained 2 µl of a dilution of target cDNA (1 : 10–1 : 10 000), 0.5 µl of each primer (20 µM), 9.5 µl milliQ water and 12.5 µl iQ SYBR Green Supermix (Bio-Rad, reference 170–8882). Samples were denatured by heating at 95 °C for 10 min prior to a 40-cycle amplification and quantification programme (95 °C for 15 s, 60 °C for 30 s and 72 °C for 20 s) with a single fluorescence measurement per cycle according to the manufacturer’s recommendations. A final 1 min extension cycle at 72 °C was performed. The PCR products were between 103 and 300 bp in length. To confirm the amplification of a single PCR product, a melting curve was obtained by slow heating from 55–95 °C at a rate of 0.5 °C every 10 s for 80 cycles with continuous fluorescence scanning. The results were normalized relative to those obtained for 16S rRNA. Quantification was based on the analysis of threshold cycle (*C*_t_) values as described by Pfaffl [51].

### Primer extension analysis

Primer extension analyses were performed as previously described by Pacheco-Sánchez *et al*. [52]. An oligonucleotide complementary to the coding strand of the *tagB1* gene (P45, Table S3) was ^32^P labelled at its 5′ ends in a 10 µl final volume that contained 1 µl 10× buffer, 10 pmol oligonucleotides, 1 µl [γ-^32^P]ATP (6000 mCi mmol^−1^), and 1 U phage T4 polynucleotide kinase. The reaction mixture was incubated for 1 h at 37 °C and 10 min at 70 °C to inactivate the kinase, and the labelled oligonucleotide was filtered through a Bio-Rad Micro Bio-Spin column to eliminate unbound nucleotide. Labelled primers were annealed to total RNA isolated as described above in a 10 µl annealing mixture that contained 2 µl 5× annealing buffer, 10^5^ c.p.m. of 5′-end-labelled primer and 50 µg total RNA template. The mixture was heated at 95 °C for 3 min, incubated at 65 °C for 5 min, and then slowly cooled to 44 °C. cDNA was synthesized by the addition of 40 µl of reverse transcriptase buffer, 1 mM of dNTPs, 0.4 U µl^−1^ of RNase inhibitor and 8 U of AMV reverse transcriptase. The mixture was incubated for 1 h at 44 °C and the reaction was terminated by adding 5 µl 3 M sodium acetate and 150 µl ethanol. The product of reverse transcription was analysed in urea–polyacrylamide sequencing gels. The gel was exposed to a GS-525 Molecular Imager (Bio-Rad).

### 5′ rapid amplification of cDNA Ends (5′-RACE)

To identify the transcription start site (TSS) of the K1-T6SS promoters, 5′ rapid amplification of cDNA ends (RACE) [53] was performed on the *tagB1* and *PP3104* genes with the SMARTer RACE cDNA Amplification kit (cat. no. 634924) from Clontech. Following the manufacturer’s instructions, total RNA was ligated to an adapter at the 5′ end, and cDNA synthesis was initiated from an oligo(dT) primer containing a 3′ adapter sequence. Gene-specific primers (P46 and P47, Table S3) were used in conjunction with the adapter primers provided to amplify 5′ and 3′ cDNA ends. The PCR products were extracted from a 1.5 % agarose gel and purified with the QIAquick PCR purification kit following the manufacturer’s instructions (Qiagen). The purified PCR products resulting from RACE were cloned into the pCR 2.1-TOPO vector (Invitrogen) as recommended. RACE products were sequenced by GATC Biotech services.

### Statistical analyses

Statistical analyses are based on the *t*-test in which two conditions are compared independently. *P*-values from raw data were calculated by two-tailed *t*-test and from ratio data to the control by one-sample *t*-test using GraphPad Prism version 8.3.0 and are represented in the graphs by ns, non-significant; *P<0.05; **P<0.01; and ***P<0.001.

## Results

### The K1-T6SS gene clusters are arranged in operons

**Fig. 2.**
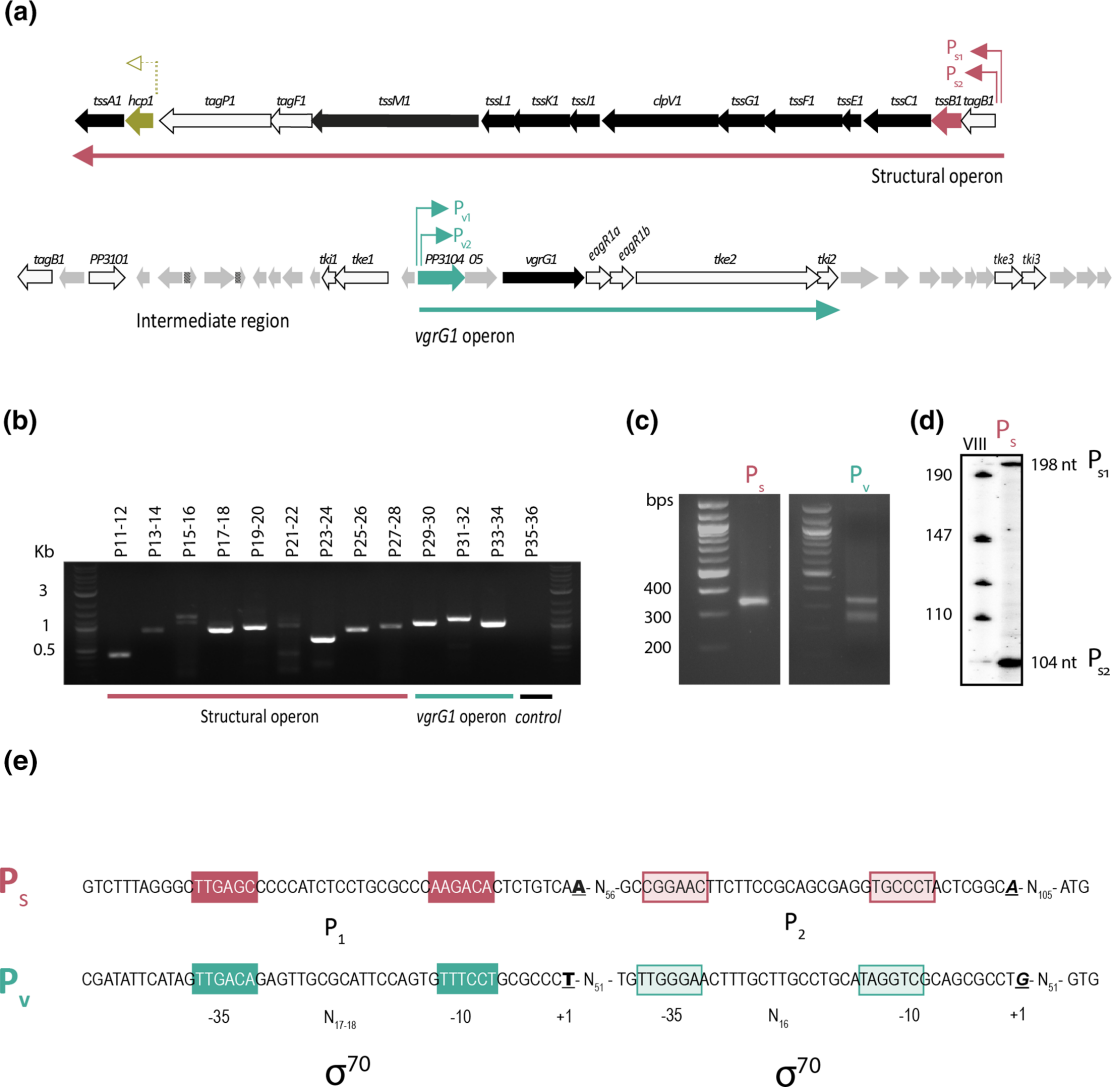
Transcriptional organization of the K1-T6SS cluster. (**a**) The 44 kb chromosomal region containing the K1-T6SS genes is shown. Block arrows represent the different genes, their relative sizes and their transcriptional orientation, with the name of the gene or the PP number (http://www.pseudomonas.com/) indicated above the arrow. Genes encoding structural components are shown in black and genes encoding accessory proteins, effector and immunity proteins, and VgrG adaptors are represented in white. Genes encoding proteins of unknown function are depicted in grey. *tssB1* and *hcp1* genes from the S operon and PP3104 from the V operon are displayed in red, gold and green, respectively. The promoter regions identified or tested in this work are shown by coloured arrows above the genes. (**b**) RT-PCR analysis to define the transcriptional units of the K1-T6SS cluster. mRNA from *P. putida* KT2440 wild-type strain grown until stationary phase was used. Primer pairs from P11 to P28 were used to amplify mRNA from the structural operon, P29 to P34 amplify the transcript of the *vgrG* operon. Primer pair P35–P36 was used as a negative control. Identification of the +1 site of the K1-T6SS structural and *vgrR* operons by 5′ RACE (**c**) and primer extension (**d**) analyses. *P. putida* KT2440 was grown in LB and samples were taken in stationary phase for total RNA isolation. (**c**) Pictures show cDNA products obtained by 5′-RACE analysis using P46 and P47 oligonucleotides (Table S3) in conjunction with adapter primers to amplify *tagB1* and PP3104 5′cDNA ends, respectively. (**d**) The autoradiogram shows the cDNA products obtained after reverse transcription of 50 µg of total RNA with the 5′-end-labelled P45 oligonucleotide (Table S3) hybridizing with the *tagB1* mRNA. (**e**) P_S_ and P_V_ promoter analyses. The *P. putida* KT2440 sequences corresponding to the regions upstream the *tagB* and PP3104 genes are shown. Nucleotides in bold, italic and underlined represent the +1 sites. The identified −10 and −35 σ^70^ binding sites are indicated. The sequences between the two promoters from the same region are in a contracted format as N_*x*_ where *x* represents the number of bases present between the two sequence segments.

The 44 kb K1-T6SS gene cluster of *P. putida* KT2440 encoding structural and accessory components comprises two predicted operons that we previously named structural and *vgrG* operon, and an intermediate region [5]. The structural operon harbours a total of 15 genes, 12 of which encode the T6SS core components (TssABCDEFGHJKLM), while 3 encode accessory elements, including TagF1, TagP1 and the recently characterized TagB1 stabilizer protein [11]. The *vgrG* operon encompasses genes encoding proteins with unknown function (PP3104 and PP3105), the VgrG1 protein and associated adapters EagR1a and EagR1b, the Tke2 nuclease toxin and its cognate immunity protein Tki2, and an additional putative effector–immunity pair (Tke3–Tki3) (Fig. 2a). The intermediate region mostly contains short ORFs that are not in the same transcriptional orientation [5], and thus it has not been considered for this study. Sequence analysis showed that most genes of the structural operon overlap and that the intergenic regions between the genes that do not overlap are smaller than 30 nucleotides (Table S4). This organization previously described by Normark *et al*. [54] indicates that these genes are arranged in a single transcriptional unit. Exceptions include the intergenic region between *hcp1-tssA1* and *tagP1-hcp1*, which are 95 and 119 bp in length, respectively. The intergenic regions between the genes of the *vgrG* operon that do not overlap are also short, ranging from 0 to 25 nucleotides (Table S4), which suggests that these genes are also arranged in a single transcriptional unit.

To test this prediction, we analysed the co-transcription of adjacent genes within the two clusters by RT-PCR using primers designed every 1–1.5 kb (Fig. S1a and Table S3) and mRNA isolated from *P. putida* cells at the stationary phase. All sets of primers were functional as determined using genomic DNA (Fig. S1b). When using mRNA, cDNA fragments of the expected sizes were obtained with all pairs of primers in both the structural (Fig. 2b, P11–28) and the *vgrG* (Fig. 2b, P29–34) clusters. This indicates that these clusters are indeed arranged in single transcriptional units (Fig. 2a). As expected, there was no amplification of a divergently transcribed region located outside the operons and used as a negative control (Fig. 2b, P35–36).

To map the promoters driving transcription of the structural (S) and the *vgrG* (V) operons, total RNA of *P. putida* cells in the stationary phase was analysed using RACE and primers complementary to *tagB1* and *PP3104* genes (Table S3). A single PCR band placed the transcriptional start site (+1) of the S operon in an adenine (A) located 121 bp upstream of the predicted translational start site of *tagB1*. This promoter was named P_S_ (Fig. 2c, left panel). Two PCR bands were obtained for the V operon, which mapped the +1 in a guanine (G) located 64 bp and a thymine (T) located 155 bp, respectively, upstream of the predicted translational start site of *PP3104* (Fig. 2c, right panel). These promoters were named P_V1_ and P_V2_. Unlike RACE, primer extension analysis of the P_s_ promoter also identified two different transcriptional start sites in this region (Fig. 2d). The lower band positioned a +1 at the same adenine as the RACE analysis. The higher band mapped a second transcriptional start site in an adenine located 215 bp upstream of the *tagB1* translational start site (Fig. 2d). These two sequential promoters of the structural operon were named P_S1_ and P_S2_ (Fig. 2).

*In silico* analyses showed that the four identified promoter regions contain σ^70^ consensus sequences located at positions −35 and −10 from the +1 (Fig. 2e). This suggests that transcription from these promoters depends on the σ^70^-loaded RNA polymerase (RNAP).

### Expression of K1-T6SS genes depends on the growth phase

The K1-T6SS has previously been described as an active system under routine laboratory conditions and in the stationary phase of growth [5, 11], a condition in which other T6SS clusters have also been reported to be active [55, 56]. To analyse the effect of the growth phase on K1-T6SS gene expression, we performed quantitative reverse transcription PCR (qRT-PCR) using mRNA from *P. putida* cells grown until the exponential or stationary phase. Representative genes from both K1-T6SS operons were used in this analysis, i.e. *hcp1* and *tssB1* for the S operon, and PP3104 from the V operon.

**Fig. 3.**
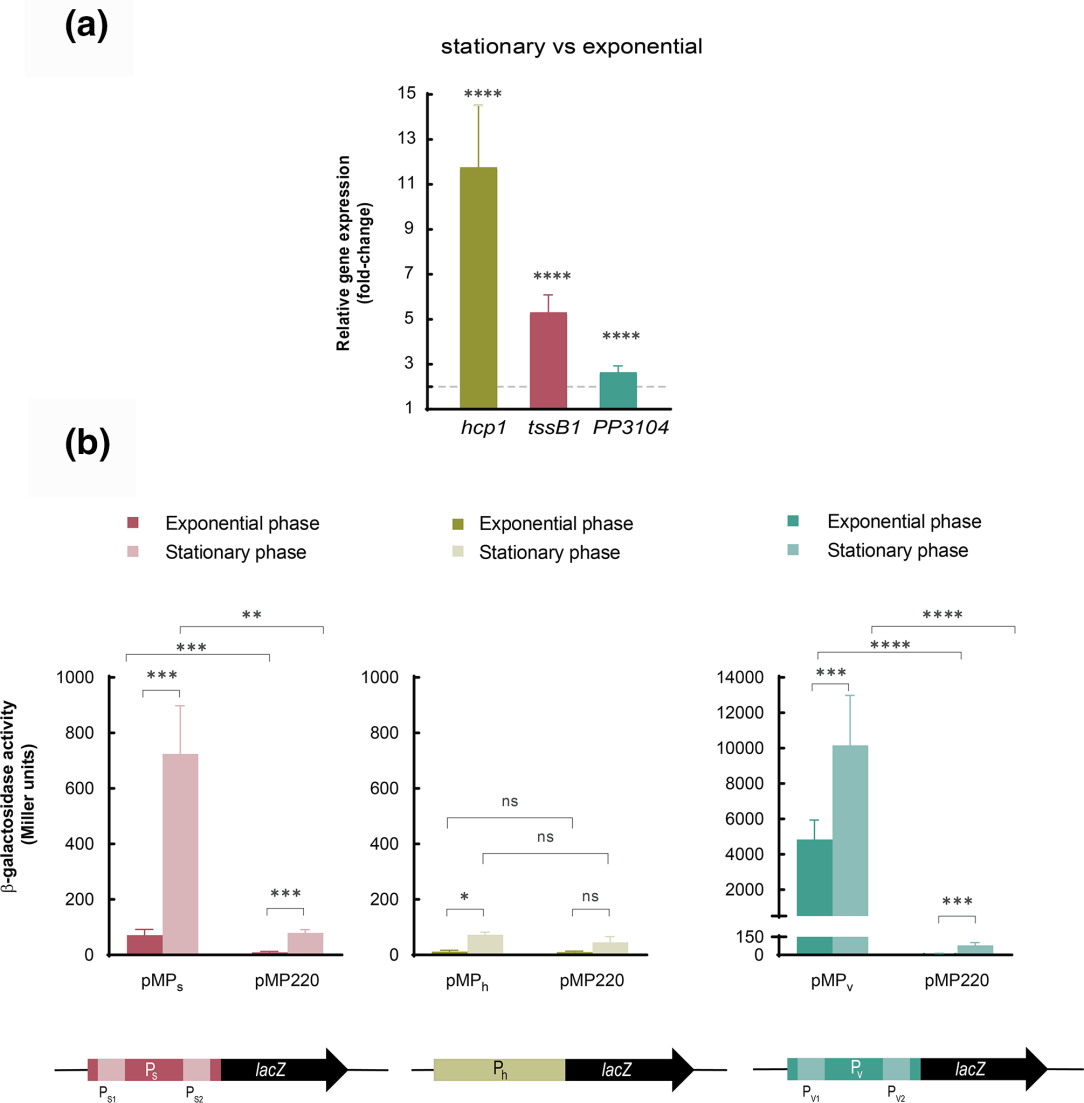
Expression of K1-T6SS genes. (**a**) mRNA levels of the indicated genes were obtained by qRT-PCR upon growth of the *P. putida* in LB until the exponential or stationary phase. The 2^−ΔΔCT^ method was used to determine the fold change range in gene expression in the stationary versus the exponential phase. Data are the means±sd from three biological replicates (*n*=3), each one including three technical replicates. *P*-values were calculated by one-sample *t*-test to a hypothetical value of 1 as described in the Methods. The horizontal dashed line denotes a fold change of 2. (**b**) β-galactosidase activity in the exponential and stationary phases of the *P. putida* wild-type strain bearing the pMP220-derived plasmids containing the indicated transcriptional fusion (P_S_, P_h_ and P_V_) to the *lacZ* gene. Strains were grown in LB until the exponential or stationary phase. Data are the means±sd from at least four replicates (*n*≥4), each one including two technical replicates. *P*-values were calculated by *t*-test analysis as described in the Methods.

Expression of the three genes was induced considerably in the stationary phase compared to the exponential phase (12-, 5- and 2.6- fold, respectively) (Fig. 3a). Interestingly, the qRT-PCR analysis showed that although *hcp1* is located at the end of the S operon and *tssB1* at the beginning, mRNA levels of *hcp1* were higher than those of *tssB1* (Fig. 3a). This suggests that *hcp1* could also be expressed from an internal promoter located upstream of *hcp1* (P_h_).

To test this hypothesis and to analyse further the expression of the K1-T6SS promoters, we constructed transcriptional fusions of P_S_, P_V_ and the putative P_h_ promoters to a promoterless *lacZ* gene using the pMP220 plasmid. The resulting plasmids, pMP_S_, pMP_V_ and pMP_h_ were introduced into the *P. putida* wild-type KT2440 strain and the β-galactosidase activity was determined in the exponential and stationary phases of growth. pMP_S_ contains the 345 bp upstream of the *tagB1* translational start site that includes the previously identified P_S1_ and P_S2_ sequential promoters (Figs 2 and 3).

In agreement with the qRT-PCR results, transcription from this promoter was practically null in the exponential phase (71 Miller units, MU) and notably induced by more than 10-fold in the stationary phase (724 MU) (Fig. 3b, left panel). Instead, the β-galactosidase activity of the pMP_V_ construct that included the 458 bp upstream of the PP3104 translational start site containing the P_V1_ and P_V2_ promoters was high in both the exponential (~5000 MU) and the stationary phase (~9000 MU) (Fig. 3b, right panel). This resulted in only 1.8-fold induction in the activity of this promoter in stationary compared to exponential phase, which was in accordance with the qRT-PCR results.

The pMP_h_ plasmid contains 509 bp upstream of the *hcp1* translational start site, which includes 378 bp of the *tagP1* 3′ coding region. The β-galactosidase activity of this construct was negligible in both the exponential and the stationary phases (12 and 73 MU, respectively), and similar to that of the empty vector control (10.6 and 45 MU, respectively) (Fig. 3b, middle panel). This indicates that there is not an internal promoter upstream of *hcp1* and that the higher detection of the mRNA from this gene is likely due to the greater stability of this part of the transcript.

### Identification of global regulators involved in the control of K1-T6SS expression

**Fig. 4.**
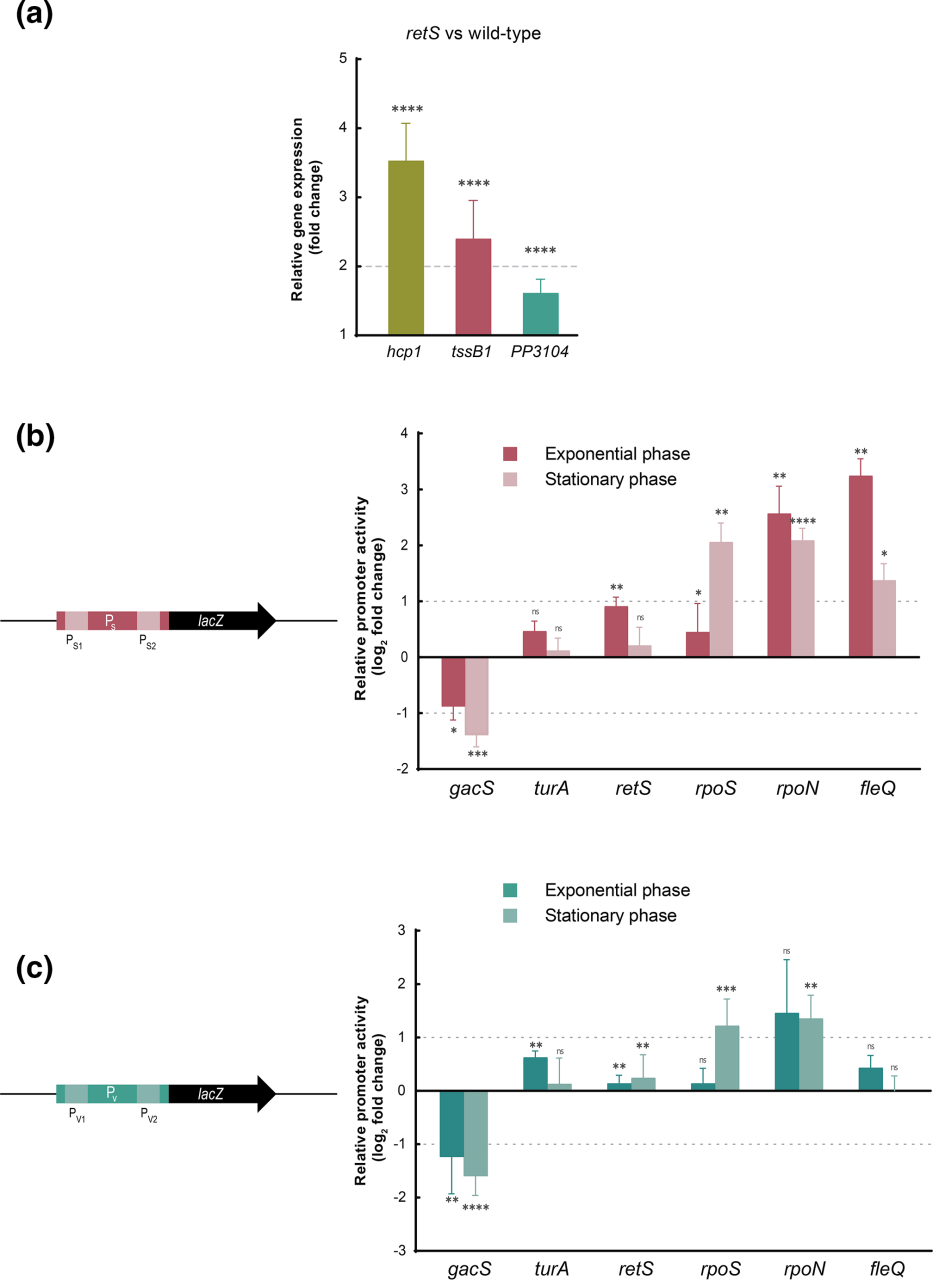
Global regulators involved in K1-T6SS expression. (**a**) mRNA levels of the indicated genes were obtained by qRT-PCR upon growth of the *P. putida* and its isogenic *retS* mutant in LB until the exponential phase of growth. The 2^−ΔΔCT^ method was used to determine the fold change range in gene expression in the *retS* versus the wild-type strain. Data are means±sd from three biological replicates (*n*=3), each one including three technical replicates. *P*-values were calculated by one-sample *t*-test to a hypothetical value of 1 as described in the Methods. The horizontal dashed line denotes a fold change of 2. The activity of the P_S_ (**b**) and P_V_ (**c**) promoters in the indicated *P. putida* mutant strain was obtained by β-galactosidase assay. Strains bearing the indicated *lacZ* fusion were grown in LB until exponential or stationary phase. Data show the fold change of the promoter activity in the mutants relative to the KT2440 wild-type strain. Data are means±sd from at least four biological replicates (*n*≥4), each one including two technical replicates. *P*-values were calculated by one-sample *t*-test to a hypothetical value of 1 as described in the Methods. The horizontal dashed lines denote a fold change of 1 in either direction represented in a log_2_ scale.

Several regulatory proteins have been involved in the control of the expression of T6SS clusters at different stages, including transcription, translational and post-translational (assembling) levels. To identify the main transcriptional regulators involved in the control of the K1-T6SS cluster, we used a battery of *P. putida* global-regulator mutants (*gacS*, *retS*, *turA*, *rpoS*, *rpoN* and *fleQ*). We first focused on the Gac signalling cascade and the *retS* mutant. This mutant has been used extensively in *P. aeruginosa* to study the activity of the H1-T6SS because this system is not produced in the wild-type strain containing RetS [1, 57]. First, we compared the expression of *hcp1*, *tssB1* and PP3104 genes in the *P. putida* wild-type versus the *retS* mutant in the exponential phase of growth by qRT-PCR. The three genes were significantly upregulated in the *retS* mutant, with fold-changes >3 for *hcp1* and >1.5 for *tssB1* and *PP3104* (Fig. 4a). Induction of the expression of the S operon in this mutant in the exponential phase of growth was confirmed by β-galactosidase using the P_S_::*lacZ* fusion (Fig. 4b). This effect was not observed in the stationary phase (Fig. 4b), which suggests that repression of the K1-T6SS by RetS occurs mainly during exponential growth. The activity of the P_V_ promoter was not affected in the *retS* mutant in either the exponential or the stationary phase of growth (Fig. 4c). However, activity of both P_S_ and P_V_ in the *gacS* response regulator mutant was considerably reduced (between two- and threefold) independently of the growth phase (Fig. 4b, c). This indicates that GacS is a positive regulator of K1-T6SS expression, and this activity is repressed only by RetS in the exponential phase of growth. Further, transcription from P_S_ and P_V_ promoters stayed almost unchanged in the *turA* mutant (Fig. 4b, c), indicating that this H-NS protein does not control K1-T6SS expression. Intriguingly, despite the fact that K1-T6SS expression is induced in the stationary phase of growth (Fig. 3), the activity of both P_S_ and P_V_ promoters was not reduced but increased in absence of the stationary σ factor RpoS (Figs 4b, c and S2). This is in accordance with the presence of σ^70^ but no RpoS consensus sequences in these promoter regions (Fig. 2e). Strikingly, the transcriptional level from P_S_ increased substantially in the absence of RpoN and FleQ (Fig. 4b), especially in the exponential phase of growth, a condition in which the system is not expressed in the wild-type strain. This suggests that these regulators mostly repress expression from the P_S_ promoter in exponential phase. The effect of RpoN is likely indirect since σ factors usually promote instead of inhibiting transcription.

### RpoN indirectly downregulates K1-T6SS expression

We next aimed to analyse the effect of RpoN on the expression of the K1-T6SS S operon. This σ factor could either promote the transcription of an unknown T6SS repressor or directly repress the transcription from the P_S_ promoter. RpoN recognizes and binds the 5′-TGGCAC-N_5_-TTGCW-3′ (W is A/T) consensus sequence located at −12 and −24 bp from the transcriptional start site [39]. The minimal recognition sequence described to be RpoN-dependent is GG-N_10_-GC [39].

**Fig. 5.**
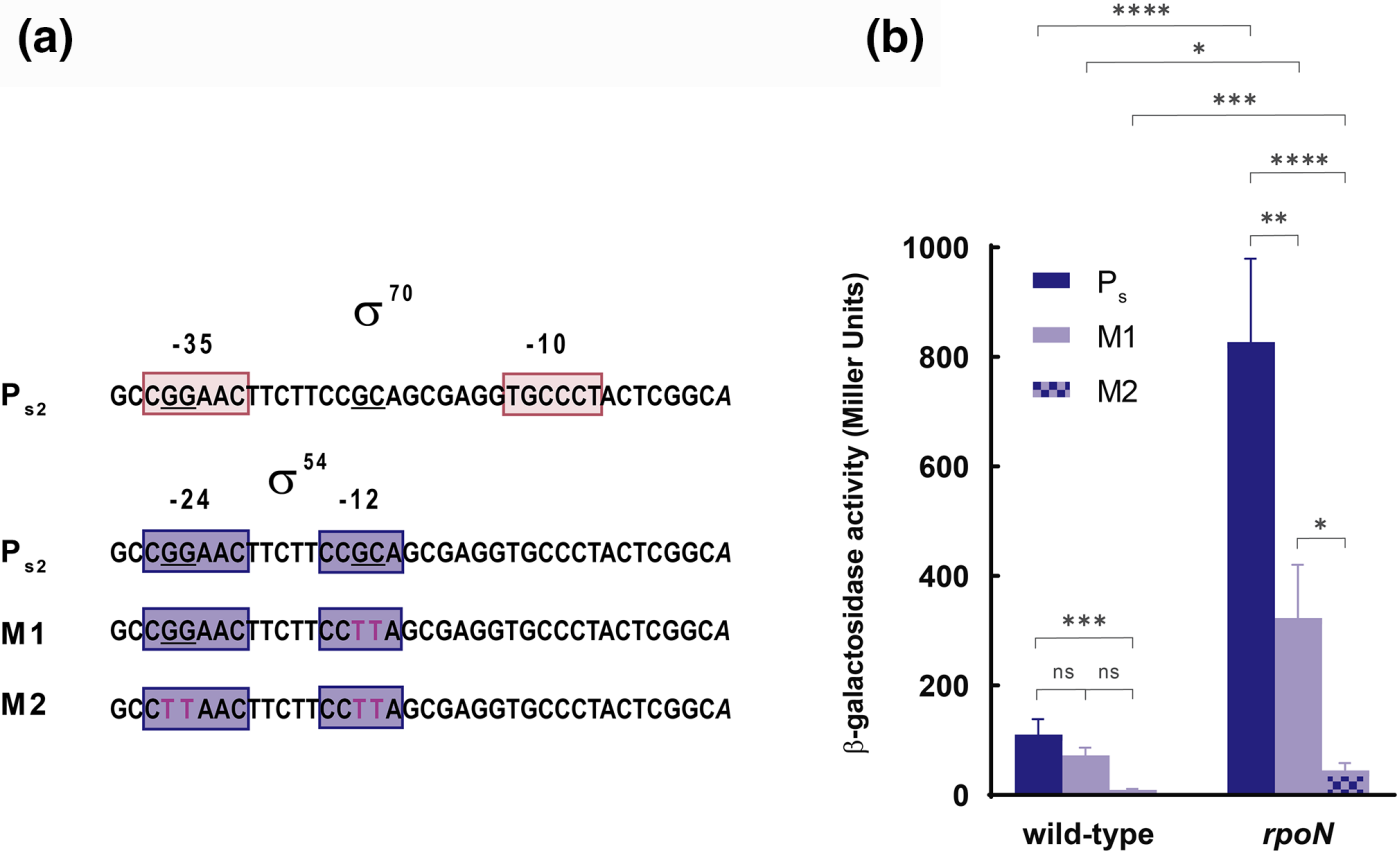
Role of the potential σ^54^ binding site in the expression from the P_S_ promoter. (**a**) The elements –10/–35 and –12/–24 are indicated, as well as the point mutations in the M1 and M2 constructs. (**b**) Data show the β-galactosidase activity of the *P. putida* wild-type and the *rpoN* mutant strains at the exponential phase of growth bearing the pMP220 plasmid containing the transcriptional fusion for the native P_S2_ and the M1 and M2 derivates to the *lacZ* gene. Data are means±sd from at least four replicates (*n*≥4), each one including two technical replicates. *P*-values were calculated by *t*-test analysis as described in the Methods.

Interestingly, we identified a potential RpoN binding site in the P_S2_ promoter that overlaps with the σ^70^ binding site (Fig. 5a). Because RpoN can stably interact with promoter sequences without initiating transcription [58], binding of this σ factor to P_S2_ could prevent the binding of the σ^70^ factor and therefore the σ^70^-mediated transcription from this promoter. It is important to note that in these cases, the so-called −12 and −24 boxes do not need to be located exactly in these positions from the transcriptional start site [58]. However, we have maintained this nomenclature of the boxes for clarity.

To test whether RpoN binds to the P_S2_ promoter, we eliminated the conserved RpoN-binding GG and GC sequences in the P_S2_ fragment included in the pMP_S_ plasmid. Because GG is part of both the −35 and −24 boxes, we produced two different constructs; M1 in which only the GC of the −12 box was changed to TT, and M2 in which both the GG and GC are changed to TT (Fig. 5a). β-galactosidase assays showed that the activity of the M2 construct was completely null in both the wild-type and the *rpoN* mutant (Fig. 5b), as expected from introducing changes in the σ^70^ -35 binding sequence. This indicates that this sequence in the P_S2_ promoter is crucial for expression of the structural operon. By contrast, the β-galactosidase activity of the M1 construct was similar to that of the wild-type promoter (Fig. 5b). This indicates that RpoN does not bind and repress the expression from this promoter. Intriguingly, the activity of the M1 construct in the *rpoN* mutant was twofold lower than that of the P_s_ wild-type construct, which could indicate the importance of the mutated region for derepression of the transcription in the absence of RpoN.

### A cell surface signalling (CSS) regulatory system neighbours the K1-T6SS cluster

**Fig. 6.**
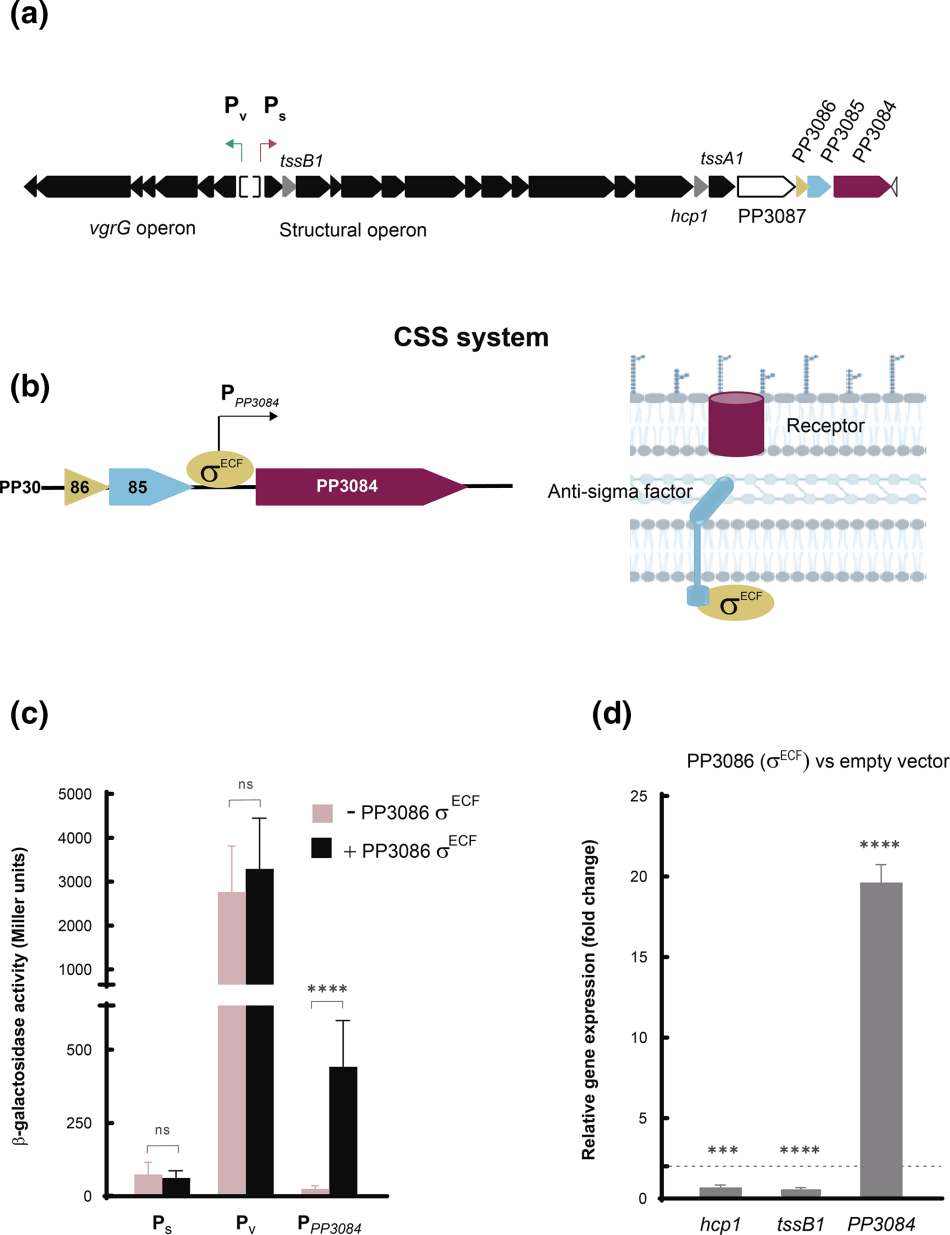
The CSS system neighbouring the T6SS cluster. (**a**) Transcriptional organization of the K1-T6SS cluster and the putative neighbouring CSS system (PP3084-86). (**b**) Close-up of the transcriptional organization of the PP3084-86 CSS system (left panel) and schematic representation of the CSS components (right panel). (**c**) β-galactosidase activity of the *P. putida* wild-type strain carrying the plasmids pMMB67EH (empty plasmid represented as −PP3086 σ^ECF^) or pMMBPP3086 (overexpressing the PP3086 σ^ECF^ factor and represented as +PP3086 σ^ECF^) and bearing the pMP220-derived plasmids containing the indicated transcriptional fusion (P_S_, P_V_ and P_PP3084_) to the *lacZ* gene. Data are the means±sd from at least four replicates (*n*≥4), each one including two technical replicates. *P*-values were calculated by *t*-test analysis as described in the Methods. (**d**) qRT-PCR analysis to determine the fold change range in gene expression of the indicated genes (*hcp1*, *tssB1* and PP3084) in the wild-type strain overexpressing PP3086 σ^ECF^ factor gene from pMMB67EH versus the empty vector. Data are the means±sd from three biological replicates (*n*=3), each one including three technical replicates. *P*-values were calculated by one-sample *t*-test to a hypothetical value of 1 as described in the Methods.

Downstream of the structural operon of the K1-T6SS cluster and in the same transcriptional orientation, there is a gene cluster encoding the three components of a CSS system (Fig. 6a). This includes an extracytoplasmic function sigma (σ^ECF^) factor (PP3086), a transmembrane anti-σ factor (PP3085) that sequesters the σ^ECF^ factor in absence of the CSS inducing signal, and a TonB-dependent outer-membrane receptor (PP3084) (Fig. 6b). CSS systems are usually activated by extracellular signals that are sensed by the receptor and produce the activation of the σ^ECF^ factor in the cytosol via the regulated proteolysis of the anti-σ factor [37]. Upon activation, the CSS σ^ECF^ factor directs the RNAP to the promoter region of target genes, which are often located in the proximity of the CSS locus and always include the CSS receptor [37, 59, 60].

To test whether this regulatory system modulates the expression of the K1-T6SS neighbour cluster, we overproduced the PP3086 σ^ECF^ factor in the KT2440 wild-type strain bearing the P_S_ or P_V_::lacZ fusions. Overproduction of σ^ECF^ factors is known to promote the expression of the σ^ECF^ regulated genes in absence of the CSS inducing signal [37, 60]. β-galactosidase assays showed that the PP3086 σ^ECF^ factor did not promote expression of the K1-T6SS cluster, since the activity of the P_S_ and P_V_ promoters was similar in the strain not overproducing (−) and overproducing (+) this regulatory protein (Fig. 6c). As a control, the activity of the promoter of the PP3084 receptor was also tested. As expected, transcription from this promoter was highly induced in the strain overproducing the PP3086 σ^ECF^ factor (Fig. 6c), which indicates that the σ^ECF^ factor is active and functional in our experimental setting. These results were confirmed by qRT-PCR. While expression of the PP3084 receptor gene increases 20-fold in the strain overproducing the PP3086 σ^ECF^ factor, transcription of the K1-T6SS hallmark genes *tssB1* and *hcp1* did not vary in the strain overproducing this σ factor (Fig. 6d). This indicates that the PP3086-3084 CSS system does not regulate production of the K1-T6SS cluster.

## Discussion

The T6SS is a complex bacterial secretion system present in many Gram-negative bacteria and used primarily to outcompete foes. The core components of T6SS nanomachines are well conserved at the structural level, and the assembly of these systems seems to be overall similar even in phylogenetically distant T6SSs [61]. A certain diversity has been described, reflected in the presence of accessory components (e.g. TagA, TagB and TagJ among others) and different forms of the core component TssA. These accessory proteins are responsible for stabilizing the sheath through different mechanisms and are related to the firing speed [11, 12, 62]. Despite these differences in the form in which the systems assemble and fire, all T6SSs arrange a similar membrane-fixed contractile tail-like structure with the ability to eject effectors into target cells. Production of these energetically expensive machines is tightly regulated [63], and it is the regulation governing its expression that varies the most.

The T6SSs are widely distributed in *P. putida* species [5, 10] that are mostly environmental strains, although some clinical isolates have been identified in recent years [64, 65]. The KT2440 strain studied in this work represents a well-established biological control agent [46]. Importantly, this strain is the model organism employed to study the role of the T6SS as a potent molecular weapon to control plant pathogens [5, 11, 66].

Here, we have studied the elements that govern the expression of the *P. putida* K1-T6SS gene cluster. The K1-T6SS contains a solo gene encoding a specific T6SS regulator, *tagF1* (Fig. 1b, purple). TagF proteins are post-translational repressors of T6SSs that, in most systems, interact with FHA proteins upon phosphorylation by a designated T6SS kinase (e.g. PpkA in *P. aeruginosa *). However, PpkA/PppA (or similar kinases/phosphatases), FHA and TagF regulatory components are not always present in T6SS systems, and any combination of those elements is possible (Fig. 1b). In fact, the K1-T6SS cluster does not contain genes encoding any kinase/phosphatase pair or FHA element and the mechanism of action of this post-translational repressor in the absence of *p*-FHA remains unknown.

In other cases, there are not genes encoding regulatory elements within the T6SS clusters, e.g. the *P. putida* K2-T6SS (Fig. 1b), but they could be located elsewhere. For example, in *Acinetobacter baumannii *, the key T6SS regulatory genes are encoded in a multidrug resistance plasmid and control the expression of the chromosomally encoded T6SS structural genes [67]. In all instances, the general regulation of the T6SS clusters is integrated into the pre-existing regulatory networks of the cell and coordinated with other important functions.

**Fig. 7.**
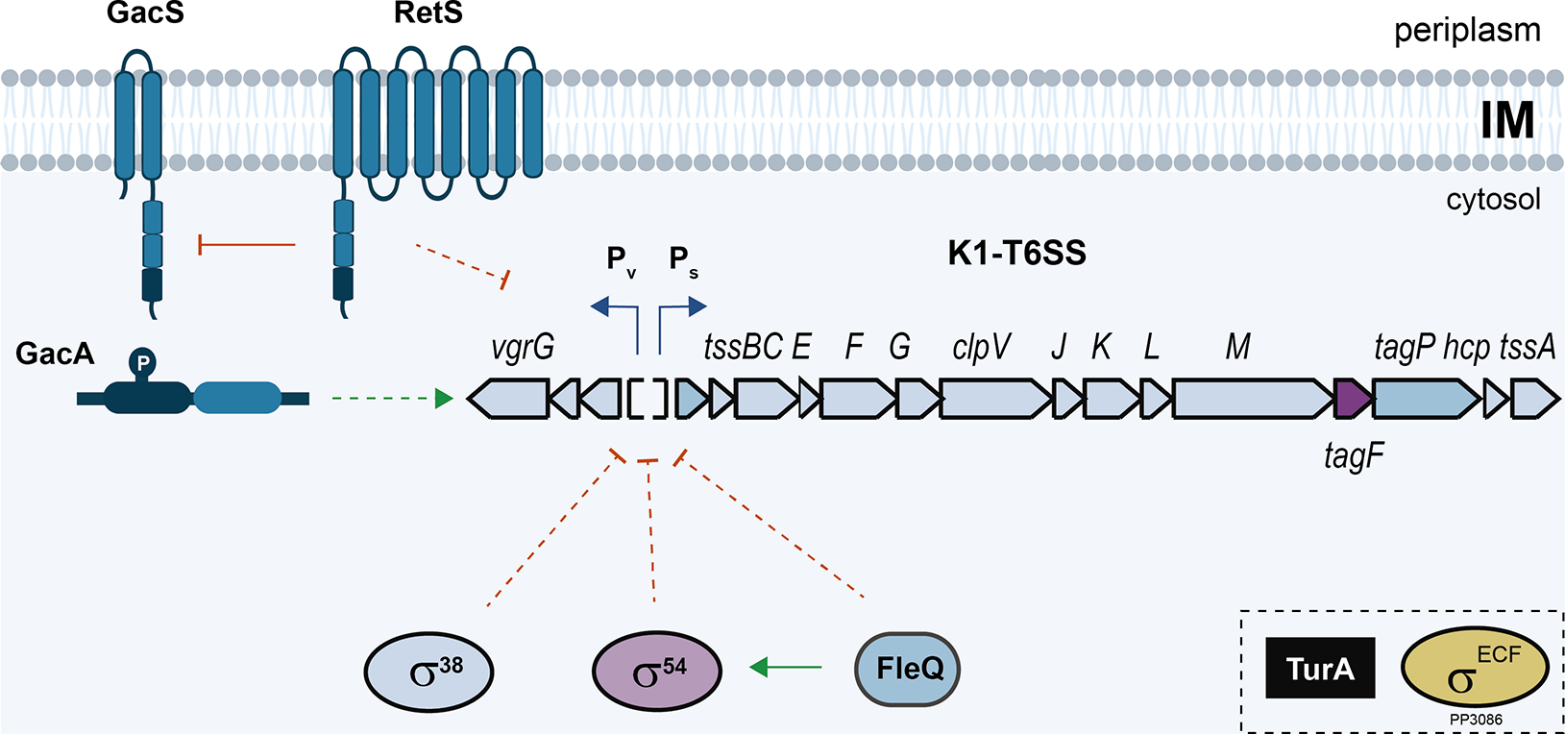
Scheme of the regulatory network governing *P. putida* K1-T6SS gene expression. The different regulatory pathways explored in this work and involved in the regulation of the *P. putida* K1-T6SS cluster are shown. Induction is represented by green arrows, while repression is shown as red bars. Discontinued dotted lines indicate an indirect effect and solid lines a direct effect.

Our analyses have shown that the *P. putida* K1-T6SS cluster is regulated in this way; its expression is driven by the housekeeping σ^70^ factor and governed by several global regulators (Fig. 7). The K1-T6SS gene cluster contains two main transcriptional units divergently expressed (Fig. 2), a common feature of T6SS gene clusters such as the *P. aeruginosa* H1-T6SS, or the K2- and K3-T6SS (Fig. 1b). The genes contained in these units are frequently grouped by function.

Accordingly, we identified a transcriptional unit containing the genes encoding the structural elements of the K1-T6SS and another called the *vgrG* operon containing the genes encoding the delivery system (the tip of the system and its associated adaptors and effectors) (Fig. 2a). Each transcriptional unit contains two transcription start sites in which −10 and −35 consensus sequences could be identified (Fig. 2), which suggests that transcription from these promoter regions is σ^70^-dependent (Fig. 2). This is in accordance with previous T6SS studies describing the σ^70^ factor as responsible for transcription of most T6SS genes [26].

In addition to the main promoter region, it is expected that lengthy operons, such as S and V, contain internal promoters to secure the expression of all components. In this regard, our semiquantitative analysis of the transcription of K1-T6SS genes by RT-PCR showed that some genes that are distant from the main identified promoter regions are highly expressed (Fig. 2b). This observation pointed to the presence of internal promoters in the K1-T6SS cluster and/or the post-transcriptional regulation of mRNA molecules that would contribute to the stabilization of these transcripts, as previously discussed for *hcp1*. These possibilities need to be further explored.

Further, our studies showed that expression of both operons of the K1-T6SS cluster was induced in the stationary phase of growth (Fig. 3). This is a relevant observation, since many *P. putida* cells in the rhizosphere have this physiological state [68] and the activation of their T6SSs in this setting might be critical to outcompete foes and eliminate plant pathogens and secure colonization and protection of the plant roots.

Other T6SSs have been observed to be induced in the stationary phase of growth, including the *Paraburkholderia phymatum* T6SS-3 [69] and the *P. aeruginosa* H2- and H3-T6SS [70]. In all these strains, it is well known that systems induced in stationary phase of growth are frequently transcribed by the stationary σ factor RpoS [71–73].

Intriguingly, despite the fact that K1-T6SS expression is induced in the stationary phase of growth (Fig. 3), the activity of both P_S_ and P_V_ K1-T6SS promoters was not reduced but increased in the absence of RpoS (Figs 4 and S2). This is consistent with the presence of σ^70^ but no RpoS consensus sequences in these promoter regions (Fig. 2e) and similar to other T6SSs that are induced in stationary phase of growth, but whose expression does not depend on RpoS [69, 70].

Likewise, we found that RpoN and FleQ factors repress the transcription of the *P. putida* K1-T6SS (Figs 4 and 7). This is in agreement with our previous observation showing that production of the Tke2 effector was considerably higher in a *rpoN* mutant [5]. Consistent with this, the number of *P. putida* cells that assemble a K1-T6SS machinery visualized by fluorescence microscopy increases from 1 % of the population in the wild-type to 10 % of the population in a *rpoN* mutant [11]. Our analyses show that the effect of RpoN on K1-T6SS expression is likely indirect because the introduction of mutations in the putative RpoN binding site identified in the promoter of the K1-T6SS structural operon did not affect its activity (Fig. 5). Therefore, both RpoS and RpoN likely modulate K1-T6SS expression by promoting the transcription of T6SS repressors, although these regulators have not been identified in this study. In *P. aeruginosa *, RpoN downregulates the expression of the gene encoding the T6SS activator GacA [74]. If this is similar in *P. putida *, RpoN could be repressing expression of the K1-T6SS cluster by downregulating the expression of the GacA–GacS cascade. Hence, the FleQ bEBP that interacts with RpoN to drive an open complex formation domain is also involved in the regulation of the K1-T6SS. This transcription factor has previously been described to activate several genes, importantly flagellar genes, and by binding to c-di-GMP, derepresses the expression of biofilm component genes [75, 76]. As expected for a bEBP of RpoN, the K1-T6SS was induced in the absence of FleQ with a slightly higher level of transcription than that observed in the *rpoN* mutant background (Fig. 4). This could indicate that FleQ negatively regulates the K1-T6SS not only through the activation of RpoN but also through an additional pathway. In fact, it has been recently described that FleQ and its partner protein, FleN, directly bind to the P_S_ promoter, inhibiting the transcription of the K1-T6SS structural operon in response to low levels of c-di-GMP [77].

In contrast, the two-component system GacS–GacA was found to activate the expression of the two K1-T6SS promoters, while the sensor protein RetS, which is known to repress the GacS–GacA cascade in *Pseudomonas* [78], represses K1-T6SS transcription (Fig. 4). The same cascade has been involved in the transcription of the *P. aeruginosa* H1-T6SS [1, 79] and *P. syringae* [80]. Among the most important functions described for the GacS–GacA TCS in non-pathogenic strains is to activate the biocontrol ability in a group of plant-beneficial pseudomonads. This activation occurs by positively controlling the production of extracellular products such as enzymes, secondary metabolites and siderophores [81]. Thus, the GacS–GacA cascade in *P. putida* could be coordinating different biocontrol strategies, such as the synthesis of classical antimicrobial compounds, the production of siderophores to sequester iron from phytopathogens and the expression and assembly of a potent molecular weapon, the T6SS.

A previous study showed that the histone-like protein TurA represses the transcription of the *P. putida* K2-T6SS [32]. Similarly, the *P. aeruginosa* MvaT protein, which belongs to the same family, silences the expression of the H2- and H3-T6SS loci [31]. However, the absence of TurA did not have a major impact on the activity of the K1-T6SS promoters (Fig. 4). This indicates that the regulation of the different T6SSs in *P. putida* is controlled, at least partially, by different global regulators. This would allow the strain to selectively activate T6SSs with different behaviours, e.g*.* the assembly and firing speed and the secreted effectors, according to the bacterium’s needs.

As expected, the structural and the *vgrG* operon are, in general, similarly regulated by the same actors (Fig. 3 and Fig. 4), guaranteeing the co-expression of the entire apparatus. The abovementioned regulators are involved in the expression of the structural components encoded in the S operon and the tip and the associated effectors encoded in the V operon (Fig. 2a). Nonetheless, the basal expression from the P_V_ promoter is 100- and 15-fold higher than the basal expression from the P_S_ promoter in the exponential and stationary phases, respectively (Fig. 3b). Since the genes encoding immunity proteins are located downstream of the effector genes within the V operon, the elevated basal expression from the P_V_ promoter could secure the expression of the immunity genes independently of the expression of the rest of the apparatus. Differentially regulating the promoter that controls the expression of the immunity genes could be an efficient mechanism of self-protection against T6SS intoxication by sister cells.

It is important to emphasize that of the seven transcriptional regulators that have been tested in this study, five are involved in the regulation of the system (Fig. 7). This provides a clear insight into the importance of controlling and monitoring T6SSs. In fact, in a recent review on the presence and absence of T6SS in bacteria, the consequences of not having a strictly regulated T6SS were discussed as a possible cause of losing this powerful antimicrobial weapon [61]. Thus, we could consider that the master regulators described in this study will allow us to precisely coordinate T6SS expression, which is necessary to outcompete foes and colonize plants, along with other important and equally energetically expensive processes to minimize energy costs and optimize fitness.

The deeper understanding of the transcriptional regulation of the *P. putida* K1-T6SS established in this study will lay the ground for the development of an improved biocontrol agent with an optimized antimicrobial weapon to outcompete deleterious plant pathogens.

## Supplementary Data

Supplementary material 1
