# Supplementary material for: Transcriptional organization and regulation of the Pseudomonas putida K1 type VI secretion system gene cluster
Source: Microbiology (Reading). 2023 Jan 27;169(1):001295. doi: 10.1099/mic.0.001295 (PMC9993120; doi:10.1099/mic.0.001295)
Supplement: Supplementary material 1 [file mic-169-1295-s001.pdf]

SUPPLEMENTARY FILE FOR

Transcriptional organization and regulation of the *Pseudomonas putida* K1 Type VI secretion system gene cluster

Patricia Bernal[1,2,3*], Cristina Civantos[1], Daniel Pacheco-Sánchez[1], José M. Quesada[1], Alain Filloux[2,4*] and María A. Llamas[1*]

[1]Department of Environmental Protection, Estación Experimental del Zaidín (CSIC), Granada, Spain
[2]MRC Centre for Molecular Bacteriology and Infection, Department of Life Sciences, Imperial College London, London, UK
[3]Departamento de Microbiología, Facultad de Biología, Universidad de Sevilla, 41012 Seville, Spain
[4]Singapore Centre for Environmental Life Sciences Engineering. Nanyang Technological University, Singapore

[*]Correspondence:
P. Bernal, Departamento de Microbiología, Facultad de Biología, Universidad de Sevilla, 41012 Seville, Spain. E-mail: pbernal@us.es
Alain Filloux, MRC Centre for Molecular Bacteriology and Infection, Department of Life Sciences, Imperial College London, London, UK. E-mail: a.filloux@imperial.ac.uk
María A. Llamas, Department of Environmental Protection, Estación Experimental del Zaidín (CSIC), Granada, Spain. E-mail: marian.llamas@eez.csic.es

This file includes:

Figure S1 and S2
Supplementary Tables 1, 2, 3 and 4
Supplementary references

SUPPLEMENTARY INFORMATION FIGURE

Figure S1

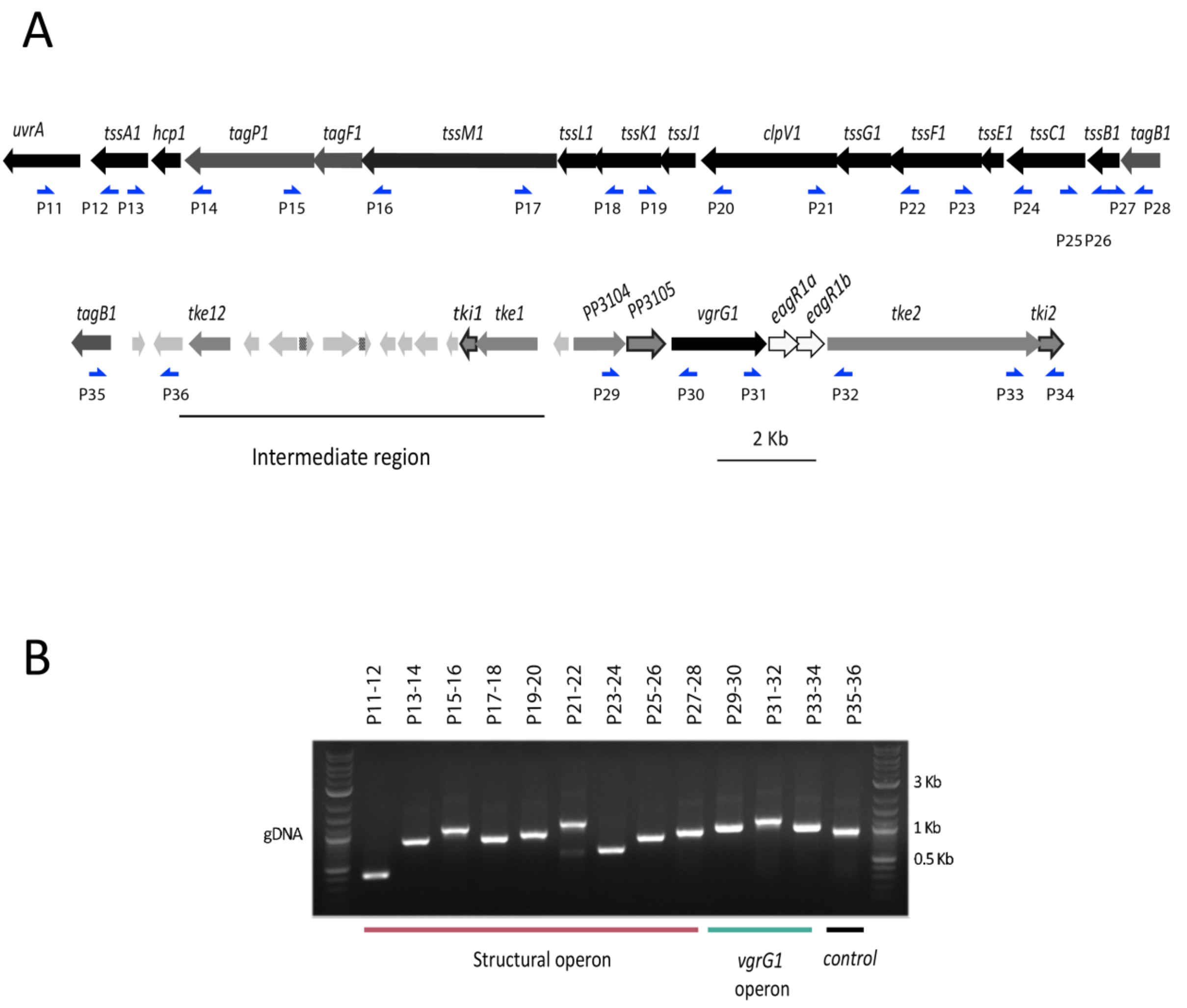

Figure S1. (A) Transcriptional organisation of the K1-T6SS cluster. The genes are located in a 44-kb chromosomal region, which includes the T6SS genes organised in 2 operons and an intermediate region. The primer pairs used to perform RT-PCR are shown in blue and the sequence can be found below in Table 3. (B) PCR analysis with gDNA of *P. putida* KT2440 as control for primer pairs used in RT-PCR analysis shown in Figure 2 to define the transcriptional units of the K1-T6SS cluster. Primer pairs from P11 to P28 were used to amplify gDNA from the structural operon and primer pairs from P29 to P34 were used to amplify gDNA of the *vgrG* operon. Primer pair P35-P36 was used as a negative control in RT-PCR analysis from a non-transcribed region. The band amplified from gDNA confirmed

the functionality of the primer pair used in this assay. (C) Primer extension analysis of total RNA of *P. putida* with primers for P_v to map the transcription start site. The DNA sequence patterned using the DNA Cycle Sequencing kit based on the Sanger method is included in the urea-polyacrylamide sequencing gel.

Figure S2

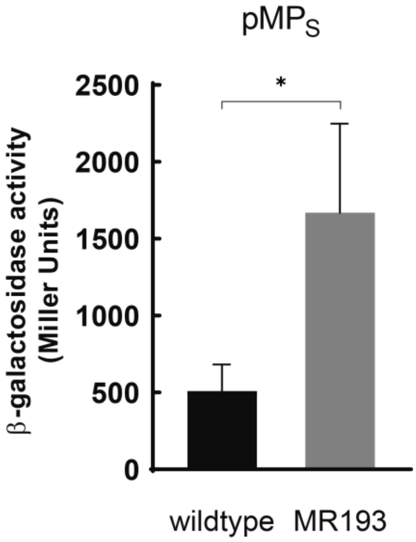

Figure S2. Expression of the K1-T6SS structural genes. β-galactosidase activity, in stationary phase, of the *P. putida* wild-type and the *rpoS* mutant MR193 strains bearing the pMP220-derivated plasmid containing the P_S transcriptional fusion to the *lacZ* gene. Strains were grown in LB until stationary phase. Data are means ± SD of three replicates, each one including two technical replicates. P values were calculated by *t*-test analysis as described in Methods.

SUPPLEMENTARY INFORMATION TABLES

Table S1. Bacterial strains used in this study.

Name	Description	Source
Escherichia coli		
DH5α	F^- *end*A1 *gln*V44 *thi*-1 *rec*A1 *rel*A1 *gyr*A96 *deo*R *nup*G *pur*B20 φ80d*lacZ*ΔM15 Δ(*lac*ZYA-*arg*F)U169 *hsd*R17($r_K^- m_K^+$) λ^-	[1]
Pseudomonas putida		
KT2440R	Rif^R	[2]
KT2440R *gacS*	*gacS*::Tn5, Kan^R	[3]
KT2440R *retS*	*retS*::Tn5, Kan^R	[3]
KT2440R *fleQ*	*fleQ::*Tn5, Kan^R	[4]
KT2440 *rpoS*	Markerless mutant	[5]
KT2440R *rpoN*	*rpoN*::*aphA,* Kan^R	[6]
KT2442 *turA*	*turA::aphA*, Kan^R Rif^R	[7]
KT2442 *rpoS* (MRB193)	Cm^R, Rif^R	F. Govantes

Table S2. Plasmids used in this study.

Name	Description	Source
pMP220	Broad host range, low copy number promoter-probe vector based on the *lacZ* reporter gene (*′lacZ*), Tet^R	[8]
pMPhcp1	*P. putida* P_{hcp1} promoter region cloned into pMP220 as a transcriptional fusion, P_{hcp1}::*lacZ*, Tet^R	This study
pMPtagB1	*P. putida* P_{tagB1} promoter cloned into pMP220 as a transcriptional fusion, P_{tagB1}::*lacZ*, Tet^R	This study
pMPvgrG1	*P. putida* P_{vrgG1} promoter cloned into pMP220 as a transcriptional fusion, P_{vrgG1}::*lacZ*, Tet^R	This study
pMPPP3084	*P. putida* P_{PP3084} promoter cloned into pMP220 as a transcriptional fusion, P_{PP3084}::*lacZ*, Tet^R	This study
pMMB67EH	IncQ broad-host range plasmid, *lacIq*; Ap^R	[9]
pMMBPP3086	pMMB67EH carrying the *P. putida* PP3086 gene and promoter region; Ap^R	This study

Table S3. Oligonucleotide primers used in this study. The "Brief description" column provides basic information on the primer design (restriction enzyme used for cloning, encoded protein, forward or reverse orientation of the primer (F or R); QRT stands for qRT-PCR primers, PE stands for Primer Extension primers and RACE stands for Rapid Amplification of cDNA Ends primers) after the / symbol it is indicated the vector where the PCR product have been cloned.

Number	Brief description	Sequence (5´-3´)
P1	*hcp1*.EcoRI.F / pMP220	GGCCGCgaattcACCATCACGCTGGAATCCG
P2	*hcp1*.KpnI.R / pMP220	GGTATAggtaccGTAAAGGGTTGCTCATGCGC
P3	*tagB1*.EcoRI.F / pMP220	CCGGgaattcGCGCACAAAATGAGAAC
P4	*tagB1*.KpnI.R / pMP220	CCTAggtaccATGTGCCTGCTTGATCGT
P5	*PP3104*.EcoRI.F / pMP220	GCTAgaattcACACCTGCCAGGCAGAT
P6	*PP3104*.PstI.R / pMP220	ATATctgcagTGGGGTCGGCTGCGGTA
P7	*PP3084*.EcoRI.F / pMP220	ATATgaattcCGCTACTGGGTAACTGTGG
P8	*PP3084*.XbaI.R / pMP220	TATAtctagaTGTGGGTGCAGAAATGAAGGTTC
P9	*PP3086*.EcoRI.F / pMMB67EH	AATTgaattcGGTCAGTGGCACGCCGCAG
P10	*PP3086*.HindIII.R / pMMB67EH	AATTaagcttGCCGCTTCGCCCAGGATCAG
P11	*uvrA-tssA1. F*	GAACGCCAACGAGGACTT
P12	*uvrA-tssA1*.R	GGCAACATGCCGTTGGAA
P13	*tssA1-tagP1.F*	CCAGCGAGAGGTCATTGAGA
P14	*tssA1-tagP1.R*	CAACCGCGAACGCAGTTGAT
P15	*tagP1-tssM1.F*	CCAGGAAGATCGCCAGGAAG
P16	*tagP1-tssM1.R*	CTTGTCGTGCGCAACTTCGG
P17	*tssM1-tssK1.F*	CAATGGCAGCAGCAACCAGA
P18	*tssM1-tssK1.R*	CAGGCCATCTGCTTCTATGC
P19	*tssK1-clpV1.F*	ACATGCCTTCCGACCACATC
P20	*tssK1-clpV1.R*	CGCAACATCGACAACATCCT
P21	*clpV1-tssF1.F*	GACCACTTCTTCGGCCAGTA
P22	*clpV1-tssF1.R*	TCAGGCCAGGTTCACAAGGCGACC
P23	*tssF1-tssC1.F*	AAGCGCAGGTGCGACAGTTC
P24	*tssF1-tssC1.R*	AAGGCCGTGACCTACTTGCG
P25	*tssC1-tssB1.F*	CATCTGCTCGGAGAGCATCG
P26	*tssC1-tssB1.R*	AATTGCTCGCACTGGAGAAC
P27	*tssB1-tagB1.F*	ATGTCCACGTCGTAGGTGAT
P28	*tssB1-tagB1.R*	ATGGACGATGCACCAATGAC
P29	*PP3104-vgrG1.F*	CCGGCTATAGTCGTGATTGC
P30	*PP3104-vgrG1.R*	GCAAGGCCAGATCGAATTCA
P31	*vgrG1-tke2.F*	TCGGCAGCATCCAGATTCC

P32	*vgrG1-tke2.R*	CTCCTGTTCCGAAGCCATCGAGCATGC
P33	*tke2-tki2.F*	ATGCGTTATGTCACTCAGGACC
P34	*tke2-tki2.R*	CCCAAGACCTGTCAACTTGAT
P35	*tagB1-PP3101.3.F*	TGCTGCGCTGTGCGCCTTGCAGGTATTC
P36	*tagB1-PP3101.3.R*	CGATGGTCGTTGAGTTGGAT
P37	*hcp1*.QRT.F	CCAGGCGAAGTACTGGATGA
P38	*hcp1*.QRT.R	GCCTTGCGGATCTTGAAGTC
P39	*tssB1*.QRT.F	AATTGCTCGCACTGGAGAAC
P40	*tssB1*.QRT.R	ATGTCCACGTCGTAGGTGAT
P41	*PP3084*.QRT.F	AGCCGTACCGCTTGCCTTTG
P42	*PP3084*.QRT.R	AAAGCGCGCCAGTACATCGG
P43	16SrRNA.QRT.F	AAAGCCTGATCCAGCCAT
P44	16SrRNA.QRT.R	GAAATTCCACCACCCTCTACC
P45	*tagB1*.PE	GCCTGCTTGATCGTCCTGAA
P46	*tagB1*.RACE	TGCTGCGCTGTGCGCCTTGCAGGTATTC
P47	*PP3104*.RACE	CGTAGGTTCTGGCTGCTACAACACTTGG

Table S4. The K1-T6SS genomic region. The table compiles information of the loci conforming the K1-T6SS clusters previously identified (Bernal et. al., 2017). Most genes in the intermediate region and downstream *tki2* are partial genes (~200 bps) and are not take into consideration in this study.

*Nucleotides between continuous genes

**Overlapping nucleotides

Locus name	Gene name	Genomic location	Continuous genes	*	**
	**Structural operon**				
PP5562	*tagB1*	3496832 - 3497596 (- strand)			
PP3100	*tssB1*	3496228 - 3496803 (- strand)	*tagB1-tssB1*	28	
PP3099	*tssC1*	3494711 - 3496213 (- strand)	*tssB1-tssC1*	14	
PP3098	*tssE1*	3494202 - 3494684 (- strand)	*tssC1-tssE1*	26	
PP3097	*tssF1*	3492382 - 3494202 (- strand)	***tssE1-tssF1***	--	**1**
PP3096	*tssG1*	3491348 - 3492418 (- strand)	***tssF1-tssG1***	--	**37**
PP3095	*tssH1/clpV1*	3488754 - 3491390 (- strand)	***tssG1-clpV1***	--	**43**
PP3094	*tssJ1*	3488007 - 3488729 (- strand)	*clpV1-tssJ1*	24	
PP3093	*tssK1*	3486667 - 3488010 (- strand)	***tssJ1-tssK1***	--	**4**
PP3092	*tssL1*	3485954 - 3486670 (- strand)	***tssK1-tssL1***		**4**
PP3091	*tssM1*	3482120 - 3485923 (- strand)	*tssL1-tssM1*	30	
PP5561	*tagF1*	3481215 - 3482123 (- strand)	***tssM1-tagF1***	--	**4**
PP3090	*tagP1*	3478723 - 3481218 (- strand)	***tagF1-tagP1***	--	**4**
PP3089	*tssD1/hcp1*	3478061 - 3478603 (- strand)	*tagP1-hcp1*	119	
PP3088	*tssA1*	3476880 - 3477965 (- strand)	*hcp1-tssA1*	95	
	**Intermediate region**				
PP5563		3498302 - 3498853 (- strand)			
PP3101	ADP-ribosyl glycohydrolase	3498998 - 3499780 (+ strand)			
PP5565		3500053 - 3500325 (- strand)			
PP5566		3500542 - 3500781 (- strand)			
PP5567		3501267 - 3501458 (+ strand)			
PP5568		3501614 - 3502312 (+ strand)			
PP5569		3502346 - 3502549 (+ strand)			
PP5571		3502704 - 3503015 (- strand)			
PP5572		3503024 - 3503305 (- strand)			
PP5573		3504009 - 3504185 (- strand)			
PP3103	*tke1*	3504568 - 3505641 (- strand)			
PP3102	*tki1*	3504275 - 3504571 (- strand)	***tke1- tki1***	--	**4**
PP5574					

	**VgrG1 operon**				
PP3104		3506683 - 3507651 (+ strand)			
PP3105		3507652 - 3508347 (+ strand)	PP3104-PP3105	0	
PP3106	*tssI1/vgrG1*	3508373 - 3510229 (+ strand)	PP3105-*vgrG1*	25	
PP5575	*eagR1a*	3510240 - 3510824 (+ strand)	*vgrG1- eagR1a*	10	
PP3107	*eagR1b*	3510805 - 3511326 (+ strand)	***eagR1a-eagR1b***	--	**20**
PP3108	*tke2*	3511333 - 3515490 (+ strand)	*eagR1b-tke2*	6	
PP5576	*tki2*	3515484 - 3515960 (+ strand)	***tke2-tki2***	--	**7**
PP5577		3516296 - 3516919 (+ strand)			
PP5578		3517095 - 3517625 (+ strand)			
PP3109		3517884 - 3518315 (+ strand)			
PP5579		3518374 - 3518853 (+ strand)			
PP5580		3519154 - 3519381 (+ strand)			
**PP3109.4**	*tke3*	3520128 – 3520717 (+ strand)			
PP5582	*tki3*	3520261 - 3520728 (+ strand)	*tke3-tki3*	11	
PP5583		3521488 - 3521847 (+ strand)			
PP3110		3521807 - 3522019 (+ strand)			
PP3111		3522028 - 3522213 (+ strand)			

SUPPLEMENTARY REFERENCES

1. **Hanahan D**. Techniques for transformation of E. coli. In: Glover DM (editor). *DNA Cloning: A Practical Approach*. Virginia; 1985. p. 109.
2. **Espinosa-Urgel M, Salido A, Ramos J-L**. Genetic Analysis of Functions Involved in Adhesion of Pseudomonas putida to Seeds. *Journal of Bacteriology* 2000;182:2363–2369.
3. **Molina-Henares MA, de la Torre J, García-Salamanca A, Molina-Henares AJ, Herrera MC, *et al.*** Identification of conditionally essential genes for growth of Pseudomonas putida KT2440 on minimal medium through the screening of a genome-wide mutant library. *Environmental Microbiology* 2010;12:1468–1485.
4. **Yousef-Coronado F, Travieso ML, Espinosa-Urgel M**. Different, overlapping mechanisms for colonization of abiotic and plant surfaces by Pseudomonas putida. *FEMS Microbiology Letters* 2008;288:118–124.
5. **Ramos-González MI, Molin S**. Cloning, sequencing, and phenotypic characterization of the rpoS gene from Pseudomonas putida KT2440. *Journal of Bacteriology* 1998;180:3421–3431.
6. **Köhler T, Harayama S, Ramos JL, Timmis KN**. Involvement of Pseudomonas putida RpoN sigma factor in regulation of various metabolic functions. *Journal of Bacteriology* 1989;171:4326–4333.
7. **Renzi F, Rescalli E, Galli E, Bertoni G**. Identification of genes regulated by the MvaT-like paralogues TurA and TurB of Pseudomonas putida KT2440. *Environmental Microbiology* 2010;12:254–263.
8. **Spaink HP, Okker RJH, Wijffelman CA, Pees E, Lugtenberg BJJ**. Promoters in the nodulation region of the Rhizobium leguminosarum Sym plasmid pRL1JI. *Plant Molecular Biology* 1987;9:27–39.
9. **Fürste JP, Pansegrau W, Frank R, Blöcker H, Scholz P, *et al.*** Molecular cloning of the plasmid RP4 primase region in a multi-host-range tacP expression vector. *Gene* 1986;48:119–131.
